# Motivation Modulates Brain Networks in Response to Faces Varying in Race and Status: A Multivariate Approach

**DOI:** 10.1523/ENEURO.0039-18.2018

**Published:** 2018-08-23

**Authors:** Bradley D. Mattan, Jennifer T. Kubota, Tianyi Li, Tzipporah P. Dang, Jasmin Cloutier

**Affiliations:** 1 Department of Psychological and Brain Sciences, University of Delaware, Newark, Delaware 19716; 2 Department of Political Science and International Relations, University of Delaware, Newark, Delaware 19716; 3 College of Business Administration, University of Illinois at Chicago, Chicago, Illinois 60607

**Keywords:** anterior cingulate cortex, behavioral partial least squares, motivation, prejudice, race, status

## Abstract

Previous behavioral and neuroimaging work indicates that individuals who are externally motivated to respond without racial prejudice tend not to spontaneously regulate their prejudice and prefer to focus on nonracial attributes when evaluating others. This fMRI multivariate analysis used partial least squares analysis to examine the distributed neural processing of race and a relevant but ostensibly nonracial attribute (i.e., socioeconomic status) as a function of the perceiver’s external motivation. Sixty-one white male participants (*Homo sapiens*) privately formed impressions of black and white male faces ascribed with high or low status. Across all conditions, greater external motivation was associated with reduced coactivation of brain regions believed to support emotion regulation (rostral anterior cingulate cortex), introspection (middle cingulate), and social cognition (temporal pole, medial prefrontal cortex). The reduced involvement of this network irrespective of target race and status suggests that external motivation is related to the participant’s overall approach to impression formation in an interracial context. The findings highlight the importance of examining network coactivation in understanding the role of external motivation in impression formation, among other interracial social processes.

## Significance Statement

This multivariate fMRI analysis examined distributed neural processing as participants formed impressions of faces varying in race and status. Across all conditions, participants reporting greater external motivation to respond without racial prejudice showed reduced coactivation in brain regions believed to support emotion regulation, introspection, and social cognition. These results suggest that external motivation may calibrate how perceivers form impressions in an interracial context, irrespective of target race. The results from this analysis raise new questions that may not have readily emerged in studies relying on traditional behavioral and univariate fMRI analyses.

## Introduction

Race remains a contentious topic in the United States and around the world. Evaluations of others based on race and other features may depend on motivations to respond without prejudice (Li et al., 2016; Mattan et al., 2018a). In contrast to individuals who intentionally cultivate a racially egalitarian self-concept (i.e., internally motivated), individuals who are motivated to avoid the social sanctions of expressing racial prejudice (i.e., externally motivated) can be especially uncomfortable when race is salient (Butz and Plant, 2009; Olson and Zabel, 2015). These motivations are frequently assessed using the internal motivation scale (IMS) and the external motivation scale (EMS; Plant and Devine, 1998). Potentially due to race-related discomfort (Amodio et al., 2006; Ofan et al., 2014), whites with high EMS scores typically engage in more effortful (albeit less efficient) self-regulation during intergroup interactions (Lambert et al., 2003; Richeson and Shelton, 2003; Richeson et al., 2003; Hausmann and Ryan, 2004; Wyer, 2007; Ito et al., 2015). High-EMS individuals also tend to avoid explicit mentions of race, focusing instead on nonracial categories or topics (Norton et al., 2006; Apfelbaum et al., 2008). In a recent fMRI study (Mattan et al., 2018a), we examined neural responses to perceived race and socioeconomic status (SES) during impression formation as a function of white perceivers’ EMS scores. Findings from this original univariate analysis indicated that EMS modulated the processing of SES (but not race) in brain regions involved in person evaluation. To gain greater insight into this intriguing set of findings, we used a multivariate approach known as behavioral partial least squares (PLS) analysis (Krishnan et al., 2011) to identify how brain networks may be modulated as a function of individual differences in perceiver motivation.

In our original univariate analyses (Mattan et al., 2018a), we found that EMS modulated responses to SES in the bilateral nucleus accumbens (NAcc) and ventromedial prefrontal cortex (VMPFC), consistent with the literature on status-based evaluations (Mattan et al., 2017, 2018b). Notably, high-EMS participants showed neural response patterns to SES that were difficult to reconcile with the largely positive evaluations of high SES (when considered independently of other dimensions) observed in the behavioral (Fiske, 2010; Varnum, 2013) and neuroimaging (Mattan et al., 2017, 2018b) literature.

In the present analysis, we used behavioral PLS analysis to examine distributed neural responses to perceived race and SES as a function of white perceivers’ EMS scores. Behavioral PLS analysis is a data-driven method that allows for the identification of one or more latent variables (LVs) that reliably account for covariance between individual differences (e.g., EMS) and distributed patterns of neural responses to conditions of interest (e.g., targets varying in race and status; Krishnan et al., 2011; Lee et al., 2011; Cloutier et al., 2017). Because this is a data-driven approach to brain–behavior correlations, behavioral PLS analysis allows for the identification of several potentially compatible LVs. One possibility is that brain–behavior correlations may differ qualitatively across conditions (Lee et al., 2011). Based on our original analysis showing EMS-related modulation of neural responses to SES (Mattan et al., 2018a), for example, EMS could correlate with increasing coactivation across a distributed network of brain regions when forming impressions of high-SES targets and with decreasing (or null) coactivation in a different network when forming impressions of low-SES targets. The converse is also possible. Although our original univariate analysis did not show a reliable relationship between EMS and localized neural responses to race (or the race-by-status interaction), it is nonetheless possible that EMS may predict distinct patterns of neural coactivation as a function of race in a multivariate analysis. For example, one study using multivoxel pattern analysis examined the neural representation of race in key regions of interest (ROIs) as white participants were assigned to one of two mixed-race groups and subsequently categorized members from both groups while in the scanner (Ratner et al., 2012). Although no effects of race were reported in the behavioral or univariate analyses, the authors did find that race was reliably decoded above chance in the visual cortex and the fusiform gyri but not in control regions (for a similar study using gender instead of race, see Kaul et al., 2011). This is particularly interesting because recent work has suggested that distributed neural responses to race are decoded more reliably in the fusiform gyri when race processing is incidental to the task (i.e., as in the present study) compared with when race processing is integral to the task (Kaul et al., 2014). A final possibility is that brain–behavior correlations are similar across all conditions (Cloutier et al., 2017). In other words, EMS could increase or decrease the overall coactivation between brain regions irrespective of face race or SES, implying that EMS influences how participants approach the task overall. Although the data-driven nature of PLS analysis obviates the need to formalize a priori ROIs, we anticipated that any latent variables would likely implicate regions involved in person evaluation (VMPFC; Cloutier et al., 2012; Mende-Siedlecki et al., 2013a; Cloutier and Gyurovski, 2014; Mattan et al., 2017, 2018b) and the regulation of prejudice (e.g., cingulate cortex, lateral prefrontal cortex; Kubota et al., 2012; Amodio, 2014; Mattan et al., 2018b).

## Materials and Methods

### Participants

Eighty-two Chicago-area men passed the initial screening. Of the 82 eligible participants, 61 completed the study. The 21 eligible participants who did not complete the study either failed to complete the on-line battery of questionnaires or were unable to schedule a suitable time for the scanning session before achieving our intended quota for this study (*N* = 60). One participant was excluded from analyses as an outlier for IMS (a control variable), exceeding 3.5 SDs from the sample mean (see Results). The final sample comprised 60 male participants (mean age, 23.8 years; SD = 4.59 years).

### Protocol

#### On-line surveys

Eligible participants completed a battery of questionnaires on-line before the day of their scan. Most of these measures were assessed for a large-scale resting-state fMRI investigation or an unrelated experiment completed immediately before the impression-formation task used for the present analysis. Although we provide an overview of pertinent measures for this report (see Experimental design and statistical analysis), full details are available in the open-access report from our previous analysis of the presented data (Mattan et al., 2018a).

#### Scanning session

On the day of scanning, participants were instructed to arrive without having consumed drugs, including caffeine and alcohol. After signing consent and imaging center paperwork, the participant was photographed and completed brief surveys. Participants were then trained on the two tasks they would complete while in the scanner. The primary experimental task involved forming impressions of faces varying in race and ascribed status. An additional task, which served as a control task for the purpose of an analysis performed for the current study, involved explicitly rating (1) the attractiveness of a series of faces depicting white actors and models and (2) the likeability of a separate set of white actor faces based on their body of work. The faces of black actors and models were not used for this control task.

Participants were first trained on the control task. They completed a practice block outside of the scanner in which they learned how they would be rating the actors and models while in the scanner. The practice block was a shortened version of the main experiment (one run with three blocks of 10 trials each), using actors and models that would not be presented in the scanner. After completing the full practice block for the control task, participants then learned about the main impression-formation task. The experimenter informed participants that the study investigated how people think of others varying in SES. SES was defined as follows: “Those who have the highest social status tend to have the most money, the most education, and the most respected jobs. Those who have the lowest social status tend to have the least money, the least education, and the least respected jobs or no job.” Following this definition, participants learned to associate colors with low- and high-status Americans (e.g., blue = low; orange = high). Status–color associations were counterbalanced across participants.

To thoroughly learn status–color associations, participants completed simple association training blocks (Cloutier and Gyurovski, 2014; Cloutier et al., 2013; Mitchell et al., 2004, 2005). In an initial block of 10 trials, participants viewed a darkened silhouette over a colored background (i.e., orange or blue: five per status level), indicating by key press whether the silhouette was low status or high status based on the background color. Participants were informed of their cumulative accuracy on each trial (mean, 98.5%). Next, participants completed a block of 10 trials (5 per status level) in which they were asked what color represents low (or high) status. Participants were again informed of their cumulative accuracy on each trial (mean, 93.4%).

Having learned the two status–color associations, participants briefly practiced the impression-formation task that they would complete while in the scanner (see Experimental design and statistical analysis). The experimenter first verbally confirmed that the participant learned the status–color associations and then explained that participants would no longer be categorizing targets as low or high in status for the impression-formation task. Instead, they would be forming quick overall impressions of male faces, taking into account all visually available information (Cloutier and Gyurovski, 2014). This was repeated for participants in the written instructions for the practice block of the impression-formation task. The procedure for the practice trial block was the same as the procedure reported for the experimental block.

Once situated in the scanner, participants first completed two fMRI runs of the control task (Mattan et al., 2018a). After this task, participants completed a brief task reminding them of the learned status–color associations and how to use the button box. All participants correctly recalled the status–color associations. After this reminder, participants completed two runs of the impression-formation task (each ∼4 min), followed by resting-state and anatomic scans, time permitting (total scan time, ∼1 h). On exiting the scanner, participants completed explicit stimuli ratings and judgments (Mattan et al., 2018a). After this block of surveys, participants were compensated and debriefed.

#### fMRI acquisition

We used a Phillips dStream Achieva 3 T system and 32-channel head coil to acquire BOLD, T2* contrast-weighted echoplanar images (EPIs). With a 2000 ms repetition time and a 25 ms echo time, we acquired 34 oblique slices using an interleaved *z*-shim acquisition protocol (Du et al., 2007). Slices were 4 mm thick with a 0.5 mm gap, a 3 mm^2^ in-plane resolution, 77° flip angle, and a 192 × 134 × 192 mm field of view. Slices were aligned to the anterior commissure–posterior commissure axis of each participant (Deichmann et al., 2003).

### Experimental design and statistical analysis

#### Design and key measures

The present analysis focuses on BOLD responses as participants formed impressions of targets varying in race and SES. We describe the impression-formation fMRI task design first, followed by the primary individual difference measures of EMS and IMS.

##### Impression-formation task

After a brief training session completed outside the scanner (see Protocol), participants learned to associate two colors with different status levels (Mattan et al., 2018a). For example, blue conveyed high status, and orange conveyed low status. Status–color associations were counterbalanced across participants.

The impression-formation task that participants completed during functional scanning adhered to a rapid event-related design (Friston et al., 1999). Trials began with a black or white male face surrounded by a blue- or orange-colored frame over a black background. After 1500 ms, the face was replaced by a white fixation of a jittered duration (i.e., intertrial interval of 500, 2500, 4500, or 6500 ms). Participants formed a quick impression of each individual by the time the face disappeared or shortly thereafter. To signal they formed an impression, participants simultaneously pressed two keys, one per index finger. Participants were informed that their responses were not meant to indicate the content of their impressions, but merely to indicate that they had formed an impression. In each run of the impression-formation task, participants viewed 60 male faces divided evenly across conditions (for details on stimulus equating, see Mattan et al., 2018a). Two reminder trials after the first and second thirds of the sequence required participants to identify the status level of a silhouette framed by either blue or orange.

Faces from all four combinations of race (black, white) and status (low, high) were interspersed in a fixed pseudorandom sequence. To optimize fMRI design efficiency (Dale, 1999), three fixed trial sequences were generated using optseq2 (Greve, 2002). For further details on trial sequence design and optimization, see the study by Mattan et al. (2018a).

##### Control task

The control task consisted of an event-related design with two functional runs. Full details on stimulus equating and counterbalancing have been reported (T.P.D., B.D.M., J.T.K., and J.C., unpublished observations). Images of actor faces and model faces were presented over two functional scans, with 30 unique white actors and 15 unique white models per functional scan. In each scan, participants rated half of the actors on attractiveness and the other half on the body of work. The models were rated only on their attractiveness.

Before each block of the control task, participants viewed a prompt indicating the evaluative judgment and target group (e.g., How attractive are these models?). All trials began with a 1500 ms presentation of a face over a black background, followed by a 500 ms fixation. After 500 ms of fixation, the fixation cross changed from white to green, prompting participants to indicate their evaluation of the actor or model. Participants responded on a scale of 1 (very attractive/likable) to 4 (very unattractive/unlikable), with key mapping counterbalanced across participants. After 1000 ms, the green fixation changed back to white and remained for an additional 1000 ms. Jittering was implemented after each trial using 0, 2000, 4000, or 6000 ms fixations.

##### Motivation to respond without racial prejudice

This 10-item measure (Plant and Devine, 1998) was administered on-line before the participant’s scheduled scan date. The EMS (Cronbach’s α = 0.874) included five items (e.g., “I try to act nonprejudiced toward black people because of pressure from others”). The IMS (Cronbach’s α = 0.764) also contained five items (e.g., “Being nonprejudiced toward black people is important to my self-concept”). Both motivations were measured on a 9 point scale from 1 = strongly disagree to 9 = strongly agree. EMS and IMS were uncorrelated in the final sample (*r*_(59)_ = 0.052, *p* = 0.694). Full details on the distributions of EMS and IMS are reported by Mattan et al. (2018a).

##### Postscan stimulus ratings

Participants completed a measure of explicit likeability for each of the 60 male face stimuli viewed in the scanner during the impression-formation task. Faces were presented with the same status-associated colored backgrounds used in the scanner. Participants rated each face on a scale from 1 = extremely unlikeable to 9 = extremely likeable.

#### Analyses of behavioral data

For the sake of completeness, we report briefly on participants’ reaction times (RTs) during the impression-formation task, simultaneously testing whether reaction times show any relationship with EMS. Using a similar approach, we also explore whether EMS predicts postscan stimulus ratings of likeability.

##### Reaction time analysis

Because of device malfunctions, RTs were not recorded from four participants. Therefore, the RT analysis included only 56 participants. Any RTs <250 ms (<0.1% of all trials) and any trials where no response was provided (1.5% of all trials) were immediately excluded from analysis. We then subsequently trimmed any remaining RTs exceeding 3 SDs from the participant’s mean RT (0.4% of all trials). RTs were then log-transformed before analysis to reduce the natural skew of RT data. To test for effects in the speed of responses during the impression-formation task, we used a linear mixed-effects model in which log-transformed reaction times were predicted by target race, target status, and the participant’s EMS score. The model included a random intercept, all possible random slopes by participant, and all possible correlation parameters.

##### Postscan likeability ratings and EMS

Using a similar linear mixed-effects model, we analyzed postscan ratings of stimulus likeability as a function of target race, target status, and the participant’s EMS score. As in the analysis of RTs, the model included a random intercept, all possible random slopes by participant, and all possible correlation parameters.

#### Analyses of fMRI data

For the fMRI data, we first summarize the preprocessing parameters and GLM parameters as reported in the original univariate analysis of these data (Mattan et al., 2018a). We then provide a detailed overview of the multivariate behavioral PLS analyses used in the present report. Additional supplemental analyses are also described.

##### Preprocessing

EPIs from each participant’s four runs (two per task) were preprocessed and analyzed at the first level using SPM8 (www.fil.ion.ucl.ac.uk/spm), facilitated by a custom suite of scripts for fMRI analysis (https://github.com/ddwagner/SPM8w). We first implemented slice-time correction (Sladky et al., 2011), using the 17th slice acquisition as the reference. Subsequently, we integrated the four repeated *z*-shim slices (Du et al., 2007). The resulting images from each participant were then unwarped and realigned to the participant’s mean EPI to correct for motion and motion-by-distortion interactions (Andersson et al., 2001). Images were subsequently normalized to the MNI template and smoothed with an 8 mm FWHM kernel (Ashburner and Friston, 1999).

##### GLM

To estimate the BOLD responses for each condition, each trial was considered as an event, and the stimulus time series was convolved with the canonical hemodynamic response function. A GLM modeled scan sequences concatenated by task as a single session with regressors for each condition. For the race-status impression-formation task, we modeled four conditions (ordered as follows: high-status black, high-status white, low-status black, and low-status white). For the control task, we modeled three conditions (ordered as follows: attractiveness ratings for actors, body-of-work ratings for actors, and attractiveness ratings for models). For both task GLMs, regressors for the key conditions of interest were followed by regressors controlling for variance associated with: (1) reminder trials; (2) low-frequency drift (i.e., a linear trend); (3) session means (1 for scan 1, 0 for scan 2); (4) six movement parameters; (5) a constant across all scans; and (6) slow fluctuation of the signal (i.e., a standard set of harmonic regressors effectively serving as a 1/128 Hz high-pass filter). Contrast images reflecting the first-level effect of each condition versus baseline were used for PLS analyses (Krishnan et al., 2011).

##### Behavioral PLS analysis

Behavioral PLS analysis is a data-driven method that allows for the identification of LVs that reliably account for covariance between individual differences on a behavioral measure (e.g., EMS) and one or more distributed patterns of neural responses to conditions of interest (Krishnan et al., 2011). In other words, the goal of behavioral PLS analysis is to find weighted patterns (i.e., the LVs) characterized by maximal covariance between the behavioral and neural datasets. A description of this method given in considerable detail can be found in previous work (McIntosh and Lobaugh, 2004; McIntosh et al., 2004; Krishnan et al., 2011; Cloutier et al., 2017). In this section, we first provide some detail on how the analysis is implemented followed by an overview of the benefits and limitations of behavioral PLS analysis.

##### Analysis parameters

**Figure 1.**
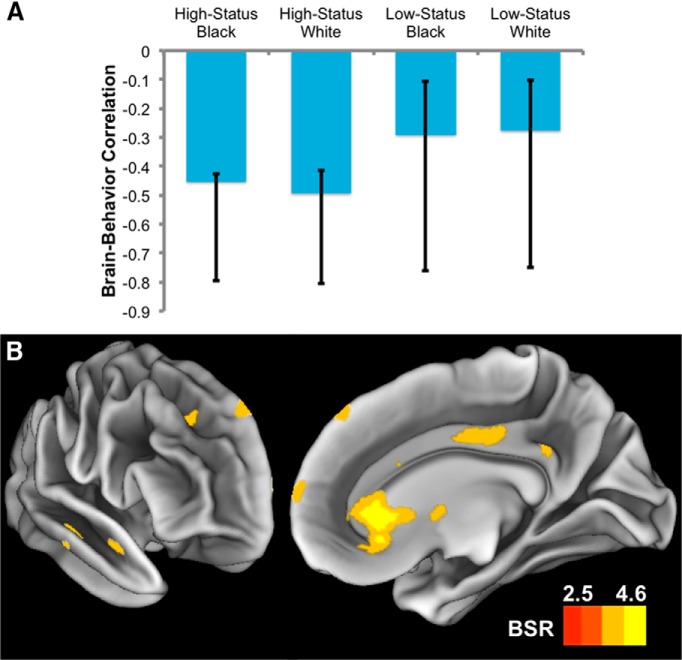
***A***, External motivation to respond without prejudice (EMS) emerged as a significant LV in behavioral PLS analysis. Brain–behavior correlations were similar across conditions. ***B***, Patterns of whole-brain activity covarying with EMS are presented on lateral–anterior (left) and medial (right) views of the right hemisphere. All voxels with BSR ≥2.5 are displayed, irrespective of their respective cluster sizes. Note that the directionality of brain activity needs to be interpreted in conjunction with the plotted brain–behavior correlations in ***A***. Increasingly positive BSRs in ***B*** indicate greater reliability of the negative brain–behavior correlations depicted in ***A***.

In the present report, we use the same analysis procedure reported by Cloutier et al. (2017) to examine the degree to which EMS predicts distributed neural responses to all conditions of interest in both the impression-formation and control tasks. To test the overall significance of each LV, a set of 2000 permuted samples was created by randomly reordering participants and condition labels (without replacement) in the voxelwise fMRI dataset, but conserving the original behavioral dataset (i.e., EMS scores). The same model used to generate the LV was subsequently applied to each permuted dataset, resulting in 2000 new covariance matrices. These covariance matrices embody the null hypothesis that there is no relationship between brain activity and behavioral data. Each covariance matrix was then subjected to singular value decomposition (SVD), resulting in a null distribution of singular values. The significance of the SVD of the original LV was ultimately assessed with respect to this null distribution. The *p* value was calculated as the proportion of the permuted singular values that exceeded the original singular value. For each significant LV, the reliability of brain–behavior correlations specific to each condition was tested using 95% confidence intervals (Fig. 1*A*). These confidence intervals were generated using a 2000-sample bootstrapping test. Because the top and bottom bounds of the confidence intervals are derived from a bootstrap distribution, it is common for these bounds to be asymmetric relative to their corresponding estimates (Efron and Tibshirani, 1986). Indeed, when the underlying distribution is sufficiently skewed, it is possible for the correlation estimate to fall outside of its bootstrapped confidence interval. We report confidence intervals derived from the standard estimation procedure built into the PLS analysis toolbox (see http://web.mit.edu/seven/src/PLS/Plscmd/pls_analysis.m).

The reliability with which each voxel contributes to the LV (i.e., the “salience” of the voxel) was also determined with bootstrapping. A set of 2000 bootstrap samples was created by resampling participants (with replacement) within each condition. Each new covariance matrix was subjected to SVD as before, and the singular vector weights from the resampled data were used to build a sampling distribution of the voxel saliences from the original dataset. The purpose of a constructed bootstrapped sampling distribution is to determine the reliability of each salience; saliences that are highly dependent on which participants are included in the analysis will have wide distributions. A single index of reliability termed “bootstrap ratio” (BSR) is calculated by taking the ratio of the salience to its bootstrap estimated SE (McIntosh and Lobaugh, 2004). A BSR for a given voxel is large when the voxel makes a strong contribution to the LV and the bootstrap-estimated SE is stable across many resamplings.

In the present study, voxel-specific BSR values were thresholded at the 95% confidence interval, corresponding to absolute BSR values exceeding 2.5. We used xjview (http://www.alivelearn.net/xjview) to identify and report the clusters of ≥20 contiguous voxels showing BSRs at or above this threshold (Tables 1, 2).

**Table 1:** Results of behavioral PLS analysis using external motivation to respond without prejudice (EMS)

Region		Cluster Size	MNI Coordinates (mm)			BSR
			*x*	*y*	*z*	
Decreased coactivation with increasing EMS						

R	Temporal pole	89	57	24	−18	3.82
R	Middle temporal gyrus	29	60	−15	−15	4.08
R	Corpus callosum	411	3	30	3	4.64
	Rostral anterior cingulate		0	33	0	3.45
	Medial orbitofrontal cortex		0	33	−13	3.35
R	Dorsomedial frontal pole	28	3	66	12	3.07
L	Temporo-occipital Junction*	21	−27	−69	15	3.41
L	Subgyral white matter	138	−24	12	27	4.42
R	Subgyral white matter	258	24	6	30	4.66
R	Middle cingulate		18	−6	34	3.37
R	Middle cingulate	39	3	−18	36	3.22
R	Dorsomedial prefrontal cortex	31	3	45	48	3.12
						
Increased coactivation with increasing EMS						
						
	N/A					

R, right; L, left. BSR indexes reliability of each cluster. All BSR ≥ 2.5; all clusters ≥ 20 voxels.

*Clusters that no longer emerge after controlling for IMS are indicated with an asterisk. Cluster subregions are reported to illustrate the anatomic extent of the cluster beyond the peak BSR.

**Table 2:** Behavioral PLS analysis results from the supplemental analysis of the control task

Region		Cluster size	MNI coordinates (mm)			BSR
			*x*	*y*	*z*	
Decreased coactivation with increasing external motivation to respond without racial prejudice (EMS)						
						
	N/A					
						
Increased coactivation with increasing EMS						
						
R	Temporal subgyral white matter	3454	45	−45	−3	6.19
	Visual cortex		0	−97	−2	5.34
	Cerebellum		−3	−76	−17	5.22
R	Putamen		30	−6	−6	3.22
R	Parahippocampal gyrus		27	3	−33	4.06
R	Amygdala		21	1	−14	4.08
R	Temporal pole		39	24	−36	4.61
L	Anterior fusiform	444	−21	3	−51	6.02
L	Temporal pole		−18	12	−37	3.66
L	Parahippocampal gyrus		−24	−2	−32	3.19
L	Subgyral white matter		−39	−14	−17	3.97
L	Hippocampus		−36	−15	−12	4.65
L	Temporal pole	108	−45	12	−48	4.01
L	Cerebellum	40	−36	−69	−42	3.11
L	Inferior frontal gyrus	381	−24	36	−3	5.44
L	Subcallosal gyrus		−18	18	−15	4.66
L	Parahippocampal gyrus	22	−21	−39	−3	3.02
L	Superior temporal gyrus	37	−69	0	0	3.71
R	Subgyral white matter	168	39	15	18	3.82
R	Inferior frontal gyrus		54	31	4	3.22
L	Insula	41	−33	−9	−18	3.41
R	Precentral gyrus	420	51	−3	21	5.19
R	Postcentral gyrus		54	−15	57	2.90
L	Middle/anterior cingulate	40	−9	24	33	3.90
L	Precentral gyrus	279	−60	6	27	4.06
L	Postcentral gyrus		−57	−9	45	3.07
R	Inferior parietal lobule	46	42	−39	27	3.92
R	Supramarginal gyrus	31	54	−21	30	3.03
L	Postcentral gyrus	66	−69	−36	51	3.57
L	Inferior parietal lobule		−57	−30	45	2.77
R	Precentral gyrus	22	33	−27	69	3.09
R	Precentral gyrus	77	6	−15	81	4.12
R	Supplemental motor area		9	−19	74	3.66

R, Right; L, left. BSR indexes reliability of each voxel. All BSR values are ≥2.5; all clusters are ≥20 voxels. Cluster subregions are reported solely to illustrate the anatomic extent of the cluster beyond the peak BSR.

##### Benefits and limitations of PLS analysis

Although other methods exist for examining changes in functional connectivity as a function of individual differences (e.g., psychophysical interaction, dynamic causal modeling), one of the primary advantages of behavioral PLS analysis relative to these methods is that behavioral PLS analysis maximizes coactivation at the whole-brain level without constraining analysis to correlations with a particular seed voxel or region (Mišić and Sporns, 2016). Behavioral PLS analysis can result in differences in brain–behavior correlations across conditions (Lee et al., 2011), albeit in a less subject-specific fashion than for more traditional analyses. This is because estimates for brain–behavior correlations are determined through a bootstrapping approach that collapses across participants. Therefore, behavioral PLS analysis can illustrate intercondition differences in at least two ways. First, confidence intervals for brain–behavior correlations in one or more conditions may contain zero. In this case, one can have little confidence that the condition containing zero in the confidence interval reliably contributes to the latent variable, unlike for the other conditions that do not contain zero in their confidence intervals. Second, confidence intervals across conditions may lie on opposite sides of zero. In this case, one can more strongly articulate a difference between conditions. Namely, conditions with positive (vs negative) brain–behavior correlations would be associated with opposite changes in coactivation in brain regions with large BSR values of the same sign (e.g., positive) as a function of the behavioral variable (e.g., EMS).

Because behavioral PLS analysis is a data-driven approach, distributed neural responses that maximally covary with the behavioral data need not be condition specific as in the preceding examples. In fact, a significant LV could reflect neural responses that correlate with the behavioral data to a similar degree for all conditions (Cloutier et al., 2017). In this case, supplemental analysis of a control task can provide additional information regarding the relative context specificity of brain–behavior correlations. For the present report, the control task served to determine whether the relationship between EMS and distributed neural coactivation in the impression-formation task, which systematically varies target race, would generalize to a different face evaluation task for which race is not a factor. Such generalization would suggest that findings from our analysis of interest (i.e., how EMS shapes neural coactivation when forming impressions of faces varying in race and status) are not task specific but rather are revealing of broader differences in the neural responses of individuals varying in EMS.

##### Supplemental PLS analyses

Because the EMS is thought to have different consequences depending on the perceiver’s IMS score (Butz and Plant, 2009), we conducted a follow-up analysis that controlled for IMS by partialing out variance in the EMS accounted for by the IMS and using the residuals in behavioral PLS analysis. Because the mean IMS score was 7.64 (on a scale from 1 to 9), the original analyses of EMS assume a high-IMS participant sample. For all analyses, the pattern of findings was similar even after controlling for IMS. As noted here and in our previous work (Mattan et al., 2018a), the limited range in IMS precludes the possibility of generalizing effects to individuals who are low in IMS (all participants scored above the midpoint of the scale).

Finally, we also conducted a task PLS analysis of the fMRI data from the impression-formation task. Task PLS analysis differs in important ways from behavioral PLS analysis for which each LV represents (1) a correlation between an individual difference (e.g., EMS) and distributed neural activity across participants and (2) the spatial pattern of voxel activations that supports that profile. In task PLS analysis, each LV represents (1) differences between experimental conditions for each participant (interpreted as a contrast) and (2) the spatial pattern of voxel activity that supports that contrast. In other words, because task PLS analysis results in brain scores at the participant level, it allows for more formal tests of differences between conditions, albeit in the absence of any individual difference variables such as EMS. In the present analyses, we used task PLS analysis to test for latent variables accounting for the relationship between the 2 (race: black, white) × 2 (status: low, high) factorial design and distributed patterns of neural responses. The same permutation and bootstrapping parameters for behavioral PLS analyses were applied to the task PLS analysis. Because results failed to return any significant LV (all *p* > 0.11), we do not further report on the task PLS analysis.

### Code accessibility

Analyses of RT and postscan ratings were conducted in R (R Core Team, 2018) using the lme4 (Bates et al., 2015) and lmerTest (Kuznetsova et al., 2016) packages. The code used to run preprocessing and GLM steps of the analysis was facilitated by SPM8 (www.fil.ion.ucl.ac.uk/spm) and a custom suite of scripts for fMRI analysis (spm8w version r5236; https://github.com/ddwagner/SPM8w). PLS analyses were conducted using a set of scripts based on an existing MATLAB-based PLS analysis toolbox (PLS Applications version 6.1311050: http://pls.rotman-baycrest.on.ca/UserGuide.htm). All code used for analysis is available from the authors on request. Analyses were performed on a linux-based server (OS, Redhat Release 7) using Matlab 2012a.

## Results

### Reaction time data

RTs were on average just under 1 s (mean RT = 977 ms; SD = 306 ms). Analyses revealed similar RTs irrespective of target race, target status, and EMS score. A marginal main effect of target status (*b* = 0.00673, SE = 0.00350, 95 CI% = [−0.000138, 0.0136], *t*_(56)_ = 1.920, *p* = 0.060) suggested a nonsignificant trend for faster responses when forming impressions of low-status (vs high-status) targets. All other effects were also nonsignificant (*p* > 0.24).

### Postscan likeability ratings

Postscan ratings of likeability revealed significant main effects of target race (*b* = 0.793, SE = 0.124, 95% CI = [0.550, 1.04], *t*_(58)_ = 6.385, *p* < 0.001) and target status (*b* = 0.413, SE = 0.106, 95% CI = [0.205, 0.621], *t*_(58)_ = 3.896, *p* < 0.001). These effects indicated greater likeability ratings for black (vs white) targets and high-status (vs low-status) targets, respectively. Consistent with the behavioral PLS analysis reported below, we observed a significant main effect of EMS (*b* = −0.175, SE = 0.0790, 95% CI = [−0.330, −0.020], *t*_(58)_ = −2.215, *p* = 0.031), with greater EMS scores associated with lower likeability ratings, irrespective of the race or status of the target. All other effects were nonsignificant (*p* > 0.19).

### PLS analysis of the impression formation task

Results revealed a significant effect of EMS as the first LV (*p* = 0.028), which explained 61.4% of the crossblock covariance. Across all conditions (Fig. 1*A*), larger EMS scores were associated with reduced coactivation in regions that form part of the emotion regulation [rostral anterior cingulate cortex (rACC)], introspection [middle cingulate cortex (MCC)], and social cognition [dorsomedial frontal pole, dorsomedial prefrontal cortex (DMPFC), and temporal pole] networks (Fig. 1*B*, Table 1). This relationship was not substantially impacted when controlling for IMS (first LV: *p* = 0.028, explaining 57.9% of crossblock covariance). Due to the similarity between these two analyses and the limited IMS variance in our high-IMS sample, all reported results are without controlling for IMS. Nonetheless, any differences that emerged between these two analyses are indicated in Table 1.

### PLS analysis of the control task

Results revealed a significant effect of EMS as the first LV (*p* = 0.025), which explained 56.7% of the crossblock covariance. Notably, only the attractiveness conditions reliably contributed to the LV: model brain–behavior correlation = 0.4235, 95% CI = [0.4568, 0.7570]; actor brain–behavior correlation = 0.1466, 95% CI = [0.0740, 0.6331]. The confidence interval for ratings of actor likeability based on body of work contained zero: brain–behavior correlation = 0.0878, 95% CI = [−0.0025, 0.4718]. In the attractiveness conditions, larger EMS scores were associated with increased coactivation in a distributed network of regions largely localized to the visual cortex, cerebellum, and sensorimotor and lateral prefrontal areas (Table 2). Note that the directionality of this effect (i.e., EMS was associated with increased coactivation between brain regions) runs in the opposite direction to that observed in the impression-formation task (i.e., EMS was associated with decreased coactivation).

## Discussion

The present findings provided the first demonstration using PLS analysis that motivation can shape the recruitment of brain networks when forming impressions of others. Specifically, increasing EMS predicted reduced coactivation of regions involved in affect regulation (e.g., rACC), introspection (MCC), and social cognition (frontal pole, DMPFC, and temporal pole) when forming impressions of faces varying in race and social status. The components of the network emerging from the impression-formation task analysis are noteworthy in several respects. We discuss each set of regions separately in the following section.

Notably, the supplemental analysis of the control task (i.e., explicit evaluations of white actors and models) provides some evidence that the negative relationship between EMS and coactivation in the aforementioned network of regions may be specific to social evaluations when race is a factor (i.e., as in the main impression-formation task). Although the supplemental analysis of the control task showed a positive relationship between EMS and coactivation of a network of regions that was distinct from the main task analysis, we nonetheless caution the reader that this difference may also reflect task differences other than the salience of race. For example, the main task involved privately forming impressions, whereas the control task required relatively more explicit and public ratings.

Beyond providing insight into the potential neural underpinnings of EMS, the present findings are also noteworthy in that the network observed in the present analysis emerged in a relatively private context. Although previous work often indicates that high-EMS individuals are typically sensitive to experimental contexts in which they believe their responses are being monitored or will be made public (Plant and Devine, 1998; Plant et al., 2003; Amodio et al., 2006), the effects of the EMS are still observed even in a private context. For example, previous studies using both EEG (Amodio et al., 2006) and behavioral methods (Plant et al., 2003) have also identified the effects of EMS on the endorsement/inhibition of stereotypes in private contexts. One possibility is that participants’ awareness that their brains were being scanned while forming impressions of black and white targets may have triggered externally motivated regulation (e.g., pipeline effects, see Plant et al., 2003). Unfortunately, these present data do not allow us to directly test the extent to which external motivation was triggered by (erroneous) beliefs about scanners reading minds. It would be interesting in a future study to examine this possibility by scanning participants who have been deceived with information that individual preferences and tendencies can be inferred from brain data versus those who have been informed about the limitations of fMRI research. Informing participants during scanning that their responses will be private (vs made public) should have a similar effect. In summary, although the mechanism requires further study, our findings add to the existing behavioral and EEG literatures, suggesting that EMS may be associated with distinct neural underpinnings even when the central threat pertaining to EMS (i.e., the potential to be exposed as harboring racist tendencies) is minimized by the private nature of the impression-formation task.

### rACC

Although the present data do not directly speak to the relationship between rACC and affect regulation, the emergence of this region in the present analysis is interesting in light of earlier work that has more directly implicated the rACC (among other regions) in the regulation of negative affect (Etkin et al., 2011, 2015) and prejudice (Amodio et al., 2006; Kubota et al., 2012; Amodio, 2014). The rACC and adjacent areas of the orbitofrontal cortex/VMPFC are thought to serve as a conduit for inhibitory signals from dorsomedial and lateral prefrontal regions en route to the amygdala (Urry et al., 2006; Johnstone et al., 2007; Etkin et al., 2011, 2015). Even in simple cognitive tasks, rACC is associated with enhanced processing of emotion-related stimuli (Kanske and Kotz, 2011) and attempts to increase emotional responses to errors under low cognitive load (Ichikawa et al., 2011). In the context of race, the rACC has been implicated in the experience of guilt after learning about one’s own implicit prejudice. More specifically, in a high-IMS score sample, rACC activity to prejudice-indicative feedback increased as self-reported guilt decreased (Fourie et al., 2014), suggesting that the rACC may have been recruited spontaneously to downregulate the negative experience of guilt in the absence of an opportunity to effectively reduce their prejudice (Amodio et al., 2007). Such an interpretation is consistent with the recent suggestion that the rACC may play a special role in implicit emotion regulation—that is, regulation arising without conscious monitoring, immediate insight, or awareness (Etkin et al., 2015).

As in previous work reporting multivariate analyses of race (Ratner et al., 2012; Kaul et al., 2014) and gender (Kaul et al., 2011) perception, response patterns differed from those we observed in our behavioral and univariate analyses (Mattan et al., 2018a). Nonetheless, we note that the rACC region detected in the present PLS analysis overlaps partially with the medial prefrontal region detected in the whole-brain analysis of the same dataset (Mattan et al., 2018a). This univariate analysis indicated that the overall larger response to high-status (vs low-status) targets reversed in high-EMS score individuals, specifically in a region involved in social evaluation (VMPFC, extending to rACC; compare with ROI analyses of VMPFC, NAcc, and amygdala). The brain–behavior correlations in Figure 1*A* are consistent with this picture (i.e., indicating numerically larger decreases in coactivation in the rACC for high-status than for low-status targets. Together, these findings suggest that EMS score may be associated with changes in both the participant’s overall approach to the task (i.e., poorer coordination between key networks previously implicated in affect regulation, introspection, and social cognition) and the participant’s sensitivity to target attributes within the task (i.e., status level). Based on the partial anatomic overlap between the findings from these two complementary studies, it will be important to more closely examine the degree to which rACC may play a unique role in supporting both task-general and target-specific effects of motivation. We believe that a multianalysis approach such as the one used for the present dataset should guide such future investigations.

### MCC

In addition to the rACC, the MCC was also part of the overall network that decreased in coactivation as a function of EMS. Although the MCC is perhaps less frequently implicated in studies on motivation or affect regulation, several studies have tied activity in this region to introspection about one’s own internal states (Herwig et al., 2010; Farb et al., 2013; Doll et al., 2016) or unpleasant emotions (Herbert et al., 2011). In the present study, we observed decreased coordination between this region and areas previously implicated in affect regulation and social cognition as a function of increased EMS. On the basis of this finding, we speculate that increasing awareness of one’s own negative internal states (vis-à-vis neural substrates in the MCC) may play an important role in circumventing the regulatory difficulties experienced by high-EMS score individuals in an interracial context (see Monteith and Mark, 2005). In any case, the present finding highlights the MCC as an important ROI in future work on external motivation to respond without racial prejudice.

### DMPFC and frontal/temporal poles

Beyond the cingulate cortex, EMS was associated with diminished coactivation in regions previously implicated in social cognition, such as the medial prefrontal cortex (frontal pole and DMPFC) and temporal pole. In general, these regions often emerge in studies of impression formation and mentalizing (Amodio and Frith, 2006). The frontal pole in particular is thought to support recently evolved aspects of social cognition including the planning and monitoring of goal-directed actions (Spreng et al., 2010; Tsujimoto et al., 2011). Recent work illustrates that the frontal pole can be divided into cytoarchitectonically and functionally distinct subregions. Meta-analyses have linked the dorsomedial subregion of the frontal pole (corresponding to the frontopolar region observed in the present study) to affective and social cognitive tasks (Bludau et al., 2014; Ray et al., 2015). For example, this region appears to be sensitive to reputational outcomes for the self and close others (Kawamichi et al., 2013). In addition, analyses of functional connectivity have revealed that the dorsomedial frontal pole is functionally connected with a number of other key regions observed in this PLS analysis, including lateral temporal cortex, rACC, and middle/posterior cingulate cortex (Bludau et al., 2014; Ray et al., 2015).

In addition to the cingulate cortex and frontal pole, we also observed EMS-related decreases in coactivation between the DMPFC and temporal pole. Previous work has implicated these regions in general impression formation (Amodio and Frith, 2006; Ames and Fiske, 2013; Mende-Siedlecki et al., 2013a,b; Li et al., 2016) and the representation of evaluative and/or stereotypic person knowledge (Gilbert et al., 2012; Li et al., 2016; Spiers et al., 2017), respectively. The EMS-related coactivation between DMPFC and the temporal pole (in addition to the rACC) overlaps considerably with the results observed in a recent study on race-based impression formation in the presence of evaluation-relevant person knowledge (Li et al., 2016). In that study, diminished activity was observed in the DMPFC, temporal pole, and rACC as high-IMS (vs low-IMS) participants formed impressions of black and white targets paired with evaluatively incongruent traits (i.e., positive and negative traits, respectively). This finding suggests that high-IMS (vs. low-IMS) individuals may be less sensitive to evaluative incongruence, resulting in diminished recruitment of regions involved in (affect-related) conflict regulation and impression formation. Notably, the present analysis indicates that these same regions (DMPFC, temporal pole, and rACC, among others) nonetheless exhibit sustained coactivation as high-IMS individuals form impressions of targets varying in race and other attributes (i.e., status). However, this coactivation between regions involved in emotion regulation and social cognition is diminished in individuals with higher levels of EMS. Together, the relationship between EMS and diminished coactivation in this social-cognitive network (in addition to regions involved in affect regulation and introspection) raises the possibility that high-EMS individuals may have been less engaged with the impression-formation task overall, despite also reporting high IMS. Future work is needed to more directly examine the relationships among coactivation in this network, task engagement, and potential mediators, such as negative affect arising from external concerns about implicit evaluative bias.

### Relevance to the neuroscience of prejudice

Previous neuroimaging work has implicated the frontal control network (including the ACC) in the regulation of prejudice in paradigms ranging from race-irrelevant spatial location tasks (Richeson et al., 2003; Cunningham et al., 2004) to race-related fear learning (Dunsmoor et al., 2016) and measures of implicit bias (Beer et al., 2008; Fourie et al., 2014). In recent reviews, the ACC [i.e., dorsal ACC (dACC)] is typically considered to reflect monitoring for conflicts between internal desires to be egalitarian and an undesirable propensity for stereotypic or prejudiced responses (Kubota et al., 2012; Amodio, 2014; Kubota and Ito, 2016; Mattan et al., 2018b). It bears mentioning that cingulate activity in the present analysis was localized to the rACC and the MCC. Although previous work on the neural substrates of prejudice regulation has focused primarily on the dACC, some have suggested on the basis of evidence from event-related potentials (ERPs) that rACC may be recruited to monitor for conflicts with external cues such as egalitarian norms (Amodio et al., 2008; Amodio, 2014). This possibility is consistent with the present finding that EMS (i.e., an external motivation) affected coactivation in a relatively rostral aspect of the ACC.

Although this is one of the first fMRI studies to examine the effects of EMS on impression formation (but see Li et al., 2016; Mattan et al., 2018a), previous work relying primarily on ERPs has long suggested that the ACC may be sensitive to perceiver motivations to respond without prejudice. Specifically, high-IMS individuals are thought to exhibit amplified conflict monitoring when race is salient (Amodio et al., 2006, 2008), even when not explicitly instructed to control their racial bias (Amodio et al., 2006). Even at high levels of IMS, increasing EMS has been observed to diminish control-related ERPs, ultimately resulting in poorer regulation of racial prejudice (Amodio et al., 2008). This is consistent with the present observation (also in a high-IMS sample) that EMS reduced overall coactivation between a collection of regions previously implicated in both affect regulation (rACC) and social cognition (frontal pole, DMPFC, and temporal pole).

Finally, it is imperative to note that the present study did not involve any revelations of prejudice; nor did it directly assess the regulation of negative affect. For this reason, it is difficult to determine what mechanism is mediating the effects of EMS on neural coactivation. Exploratory analyses of postscan stimulus ratings indicated a significant negative relationship between EMS and ratings of target likeability irrespective of target race or status, providing indirect support for the notion that high-EMS participants may be less predisposed to like others in the context of this interracial impression-formation task. The reason for this decline in likeability ratings as a function of EMS is unclear. It is possible that forming impressions of any individual in an interracial context is particularly uncomfortable for individuals with high EMS scores (Norton et al., 2006; Apfelbaum et al., 2008; Butz and Plant, 2009; Olson and Zabel, 2015), resulting in lower overall likeability ratings. In summary, it will be important for future work to examine additional behavioral correlates of EMS to triangulate more precisely what psychological mechanism underlies the relationship between individual differences in EMS and the pattern of neural coactivation observed in the present study. Consistent with existing evidence that high EMS affects neural control mechanisms in participants concerned about appearing prejudiced (Amodio et al., 2006; Ofan et al., 2014), one possibility is that externally motivated concerns (e.g., about the scanner detecting one’s prejudice) may have diminished effective regulation of negative affect arising from conflicts between racial/class bias and intentions to form unbiased impressions (Fazio and Hilden, 2001; Devine et al., 2002). Alternatively, EMS may be associated with a diminished awareness of and/or propensity to regulate negative affect in the first instance. Further research is needed to differentiate between these and other possibilities.

### Conclusion

Using PLS analysis, we found that EMS diminished coactivation between brain networks previously implicated in affect regulation, introspection, and social cognition as high-IMS white perceivers formed impressions of targets varying in race and status. Notably, this EMS score-related decrease in coactivation was observed in all conditions, suggesting that EMS was associated with the way participants approached the impression-formation task as a whole rather than their responses to attributes of the targets, such as status (but compare with Mattan et al., 2018a). The emergence of the rACC in the present analysis is noteworthy in light of previous work that has more directly examined the role of this region in prejudice regulation (Amodio et al., 2008; Kubota et al., 2012; Amodio, 2014). Moreover, together with the previous univariate analysis of the same dataset (Mattan et al., 2018a), the present analysis suggests that the rACC may uniquely contribute to both task-specific and target-specific effects of motivation to respond without racial prejudice. Finally, the current findings also raise new questions regarding the relationship between self-reported levels of EMS and the psychological and neural mechanisms of prejudice regulation.

In conclusion, the present PLS analysis provides insight above and beyond what was previously obtained using univariate analysis (Mattan et al., 2018a), suggesting that EMS leads to decreases in coactivation in regions previously implicated in emotion regulation, introspection, and social cognition. Although the precise mechanism underlying this EMS-related decrease in coactivation across this network requires further study, we believe that this network and multivariate approach will be a fruitful starting point for research into the neural substrates of previously established relationships among EMS, race-related discomfort (Norton et al., 2006; Apfelbaum et al., 2008; Butz and Plant, 2009; Olson and Zabel, 2015), and prejudice regulation (Lambert et al., 2003; Richeson and Shelton, 2003; Richeson et al., 2003; Hausmann and Ryan, 2004; Wyer, 2007; Amodio et al., 2008; Ito et al., 2015).

## Synthesis

Reviewing Editor: Philippe Tobler, University of Zurich Decisions are customarily a result of the Reviewing Editor and the peer reviewers coming together and discussing their recommendations until a consensus is reached. When revisions are invited, a fact-based synthesis statement explaining their decision and outlining what is needed to prepare a revision will be listed below. The following reviewer(s) agreed to reveal their identity: Macià Buades-Rotger.

Mattan and colleagues present an fMRI study using a Partial Least Squares (PLS) multivariate analysis. White male participants viewed pictures of black and white male faces that had been ascribed a low or high socioeconomic status in a 2x2 design (Face Race: White, Black, Socioeconomic Status: High, Low). Participants were instructed to make a judgment about the face in the scanner. Participants scoring high in a measure called EMS which quantifies the degree to which individuals are externally motivated to respond without racial prejudice showed reduced co-activation across all conditions (regardless of race or ascribed social status of the face) in brain regions putatively involved in emotion regulation (rostral cingulate cortex) and social cognition (temporal pole and medial prefrontal cortex). The authors conclude that the reduced activity in these regions may help explain why high-EMS individuals are less effective at regulating their prejudice and engaging in interracial interactions.

Both reviewers found the rationale for this study appropriate and the methodology sound. Although this same dataset was previously analyzed with a mass univariate approach, the use of a multivariate technique provides a novel data-driven estimate of connectivity during social-status estimation. However, both reviewers also raised the crucial point that in its current form, the manuscript does not make any real contribution to the literature, due to the fact that there are no differences between conditions. As a consequence, no conclusions can be drawn from the paper and it is entirely unclear what the reader learns from the paper, which if unaddressed will preclude publication in eNeuro. However, the reviewers make helpful suggestions of how to address this point (major concern 1) and raise several other concerns. If you feel that you can thoroughly and satisfactorily address all concerns, we would be happy to consider a revised version of your manuscript.

* We thank the editor and reviewers for their careful evaluation of our work. We read this synthesis of reviews with great interest and believe that we were able to satisfactorily address the major concerns raised in the review process. As a result of these extensive revisions, we believe that the quality and contribution of the manuscript is much improved.

Major concerns:

1. Interpretation: why would EMS affect the judgement of faces regardless of their race? As the significant brain activity is observed across all experimental conditions, there is no way of being certain that the brain activity is related to the task-at-hand without a control condition for comparison. By extension, statements such as "The present findings provided the first demonstration using PLS that motivations can shape the recruitment of brain networks when forming impressions of others." and sentences stating that the present results "corroborate" the rACC's role "in the regulation of prejudice" or "previous behavioral and neuroimaging work indicating that EMS is associated with greater race-related discomfort" are simply not justified by the results. Perhaps high-EMS individuals do recruit these brain networks less, but there is no way of knowing in the current paradigm whether this brain activity has to do with the formation of impressions. Moreover, for the statements to hold, there should indeed be differences between the processing of black and white faces. The reviewers suggest several options for how to address this concern:

a. The authors could run a similar PLS analysis on the other task that subjects performed in the scanner (assuming it was unrelated to impression formation), or with resting-state data if they have any. If the network identified in the impression formation task emerges to a statistically significant lesser degree in these other data, their claim would be supported. This would lengthen the paper, but it would also strengthen it.

b. Contrast task blocks vs baseline. If the EMS-related network emerges for task relative to rest blocks, their main claim would be partially supported. Of course, here we have the problem of carryover effects, which is made worse by the fact there are no null trials, and that some inter-trial intervals are very short and the BOLD signal doesn't go back to baseline.

c. It is conceivable that EMS should correlate with reaction times during impression formation (maybe high-EMS subjects respond more quickly to "hide" their prejudice?). The authors could check whether that is indeed the case. One could then inspect for brain-behavior correlations with reaction times, and see whether a similar network emerges. Perhaps that could be achieved by running the PLS analyses with reaction times instead of EMS as a behavioral variable, or maybe by extracting a global measure of network integrity and correlate it post-hoc with reaction times across participants.

If none of these works, the paper would have to be completely rephrased (removing any reference to impression formation or judgment making) and a different contribution would have to be worked out.

* We thank the reviewers for pointing out this potential limitation of the original manuscript and for the helpful suggestions on how to remedy it. After a thoughtful consideration of the three preceding recommendations, we decided that the first option would be best suited to address this important question in the context of our study and could potentially help support our interpretation of the central finding reported in the original submission. We therefore examined how EMS predicted distributed neural co-activations during a “control task” that required explicit ratings of faces on attractiveness and likeability (Dang, Mattan, Kubota, & Cloutier, in preparation). In further detail, participants viewed faces of White actors (intended as familiar targets) or White models (i.e., unfamiliar targets). Participants rated both actors and models on their attractiveness. For actors only, participants rated these targets for likeability vis-à-vis their body of work. Participants completed separate blocks for each combination of occupation and rating. By running the same behavioral PLS analysis on a somewhat similar experimental design (viz., participant responding to various conditions of faces) we believe we are now able to adequately address the concern that our reported EMS effects may be task independent and not tied to forming impressions of targets varying in race and status.

* Importantly, we found completely different patterns of results from this additional analysis. Although the results of this second PLS analysis do identify a significant relationship between EMS and a distributed network of brain regions, the network in question is distinct from that observed during the impression formation task of interest. This network differs from the network that emerged for the race-status impression formation task in several important respects. First, only ratings of attractiveness (either for models or actors) reliably contributed to the latent variable (confidence intervals for ratings of actor likeability based on body of work contained zero). Recall that all conditions contributed to the latent variable in the primary PLS analysis of the impression formation task. Second, EMS positively predicted co-activation across this network. This runs contrary to what we observed in the impression formation task analysis reported in the original submission, which showed a negative relationship between EMS and network co-activation. Third, the network revealed in the analysis of the “control task” was largely localized to the visual cortex, cerebellum, sensorimotor, and lateral prefrontal areas (see page 19 for results). Although it is unclear from an a priori standpoint why EMS would predict any network co-activation during evaluations of the attractiveness of actors and models, and we would not want to interpret these intriguing findings at this stage, results from this “control task” analysis nonetheless indicate that EMS shows a qualitatively distinct relationship in the context of our race-status impression formation task compared to a different face evaluation task (i.e., the actor-model ratings task). As stated in the original submission, EMS was associated with co-activation in regions during the race-status impression formation task that have previously been implicated in emotion regulation and social cognition. Notably, these same regions are absent in the analysis of the control task in which participants rated only White actors and models.

* Finally, we agree with reviewers that differences in how EMS predicts network co-activation as a function of target race would provide more compelling neural evidence of prejudice regulation. However, this pattern is not bourn out by these data. If anything, the effect of EMS on network co-activation appears to be more sensitive to target status than target race. Although the brain-behavior correlations in Figure 1A indicate numerically larger decreases in co-activation for high-status than for low-status targets, behavioral PLS does not afford a formal means of testing the reliability of differences in brain-behavior correlations across conditions. This is because estimates for brain-behavior correlations are determined through a bootstrapping approach that collapses across participants.

* Regardless of whether brain-behavior correlations in the present analysis differ by status level (or race), we believe that the PLS analysis of the control task and the original univariate analysis (Mattan et al., 2018) now provide sufficient support for our interpretation of the findings. For both of these points, we are indebted to the reviewers' suggestions. Taken together, our findings suggest that EMS is associated with changes in both the participant's overall approach to the race-status impression formation task (i.e., poorer coordination between key networks involved in self-regulation and social cognition that is specific to the interracial context) and the participant's sensitivity to target attributes within the task (i.e., status level of the individual targets). Based on the partial anatomical overlap between these two complementary effects (see discussion of the rACC in response to reviewer comment 10), it will be important to more closely examine the degree to which rACC may play a unique role in supporting both task-general and target-specific effects of motivation.

In addition to the three options above, the referees discussed also the following related points. For (e) and (f), clarification should be provided in the paper in any case. All three have some potential to help addressing the concern:

d. Perhaps some other contrasts could be more revealing of the relation between high- and low-EMS participants. Moreover, there's a large subject pool, so perhaps blocking participants into high- and low-EMS and seeing if similar network differences are observed with task conditions might help?

* We thank the reviewers for this suggestion. We hope that the additional analysis reported above will sufficiently address the reviewers' concerns. Furthermore, although conducting a median split can sometimes provide a useful depiction of group differences, we fear that doing so in this context would be incompatible with the use of the behavioral PLS analysis we utilized to examine the relationship between a continuous behavioral score and neural responses and potentially reduce our power to detect these relationships primarily due to the reduction in the inherent variability of the predictor variable (Irwin & McClelland, 2003).

e. It wouldn't necessarily be real support, but the paper would benefit greatly from including some details from the explicit judgments recorded from participants outside of the scanner. Were these just free form responses? Or were they answering on any sort of scale? The former would be tough to do anything with, but if some consistent differences could be identified for high-EMS individuals, that would help the narrative (provided the interpretation of the fMRI findings were rephrased as well).

* We thank the reviewers for this helpful suggestion. After scanning, participants completed three explicit judgment tasks involving the stimuli that were used in the race-status impression formation task. Two of these tasks (trust game and status recall) were intended as pilot data for an ongoing project on the joint influence of race and status on financial trust decisions. Another task assessed the likeability of each target from the race-status impression formation task. We propose to report on analyses of the likeability ratings due to the relevance of this construct to impression formation and our desire to avoid reporting data that may later be published in our ongoing project on financial trust decisions. As reported in the original analysis of these data (Mattan et al., 2018), the post-scan explicit judgments of face likeability were provided on a 9-point scale. Information on this scale is now provided in the method section on page 10. In line with the reviewers' recommendation, we now provide an additional supplemental analysis testing for any relationship between EMS and these likeability ratings (see pages 11). In line with the pattern that emerged in our PLS analysis of the impression formation task, we found that EMS scores predict lower ratings of likeability, irrespective of target race or status (see pages 16-17 for results). In the spirit of the reviewers' central recommendation for this revision, we would ideally compare likeability ratings of targets from the impression formation task to ratings of the actors and models from the control task. Unfortunately, likeability ratings that were recorded during scanning were tied to each actor's body of work. We did not collect likeability ratings for the models inside or outside the scanner. Therefore, we report only the relationship between EMS and likeability ratings for the targets from the race-status impression formation task.

f. How stable are the non-parametric p values? The trial count seemed pretty low (120 trials total, 30 each condition if I'm reading the methods right?) If these are based on only 1000 permutations, perhaps the task related PLS analysis would benefit from more permutations to build the null distribution.

* We thank the reviewers for this observation and agree that the analyses may benefit from the use of more permutations and bootstrapping. We therefore increased each of these parameters from 1000 to 2000 for all analyses (both the behavioral and task PLS analyses). Using 2000 permutations/bootstraps meets or exceeds the numbers reported in most published studies. The results are largely the same when twice as many permutations are used, although we note that the extent of the clusters reported in Table 1 appears to have sharpened by a few voxels for each cluster. Because of these small improvements in the stability of the results, we therefore report the results of the new analyses using 2000 permutations/bootstraps.

* The task PLS analysis failed to return any significant LVs after 2000 permutations. This was the case even after an exploratory analysis using 10000 permutations.

2. Introduction: The authors sufficiently justify the use of PLS relative to univariate analyses. However, it is not clear what the advantage of PLS is over simpler methods such as e.g. regression analyses on voxel-to-voxel connectivity maps. Can the authors comment on this?

* We agree that it is important to provide a context for one's selection of analysis methods, particularly when they are not yet frequently utilized. Although other useful methods allow for examining changes in functional connectivity as a function of individual differences (e.g., psychophysical interaction, dynamic causal modeling), the primary advantage of behavioral PLS is that it can be completely data driven and that it maximizes co-activation at the whole-brain level without constraining analysis to correlations with a particular seed voxel or region (Misic & Sporns, 2016). Although we believe that providing detailed comparisons of these different methodological approaches is beyond the scope of our study, we have added a similar comment on the relative utility of PLS to the method section at the top of page 13.

3. Methods: 82 participants passed initial screening but only 61 completed the study. What happened with the other 21 participants? Why did they not complete the study?

* Most of these 21 eligible-but-unscanned participants were unable to schedule a scan time that suited their schedules prior to the conclusion of the study. Additionally, some participants never completed the required online battery of measures. We now provide further information on these participants on page 6.

4. Methods: Where did the faces used as stimuli come from? Do they come from a scientifically sound database matched for any confounding factors?

* We thank the reviewer for bringing this question to our attention. The faces in this study come from the Chicago Face Database (Ma et al., 2015). As reported in our recently published paper (Mattan et al., 2018), the face sets used in this study were equated on a number of dimensions. We explicitly refer the reader to our previous paper at the bottom of page 9. For the convenience of the reviewers, we summarize the relevant excerpt from that paper's online supplement here:

* Thirty Black and 30 White faces were selected from a pool of 372 Black and White faces from the Chicago Face Database (Ma, Correll, & Wittenbrink, 2015) and other published work (Meissner, Brigham, & Butz, 2005). Original photographs were from the shoulders up. Prior to pre-testing, the following inclusion criteria were used: (1) direct eye gaze, (2) upright head position, (3) no glasses, and (4) no piercings. Any jewelry or piercings were removed using Adobe Photoshop, where possible. All images were equated on contrast and luminance using the SHINE toolbox (Willenbockel et al., 2010), and backgrounds were changed to light gray.

* To ensure that the resulting four groups were as similar as possible, faces were equated using independent ratings collected on Amazon Mechanical Turk. Separate groups (N = 24-41) of participants rated the large pool of 372 faces for race, expression, trustworthiness, dominance, attractiveness, likeability, and threat.

* After making an effort, to equate face sets on as many dimensions as possible, we ultimately selected 30 White and 30 Black faces (15 per status level) for the present experiment. Separate 2 (Race: Black, White) × 2 (Group: blue, orange) ANOVAs on mean ratings for each of the 60 faces confirmed that the four face groups were equated on several dimensions. Overall, 97.1% of faces were correctly categorized as White or Black; the four face groups were similarly high in racial categorization accuracy. Across all conditions, 59.9% of faces were most frequently perceived as presenting a neutral expression; the four face groups did not differ in the frequency of neutral expressions. However, other emotions were more frequently perceived for some faces, including anger (13.7% of all faces), happiness (12.4% of all faces), and sadness (14.0% of all faces). Importantly, the four face groups did not differ on any of these less frequently identified emotions. Expressions were overall low in intensity (M = 5.58, on a scale from 5 to 9); the four face groups did not significantly differ in their intensity ratings. Overall, faces were rated (on scales from 1 to 7) as similarly low to moderate on the remaining dimensions of trustworthiness (M = 3.08), dominance (M = 4.02), attractiveness (M = 2.87), and likeability (M = 3.39). The four face groups did not differ in their ratings on any of these dimensions.

* Due to the number of dimensions assessed, we were unable to equate face groups in terms of threat. In our final set of face stimuli, Black faces (M = 3.75) had significantly lower threat ratings than White faces (M = 4.02), F(1,56) = 7.67, p = .008, ?p2 = .120. The blue-framed faces (M = 3.77) were also rated as significantly more threatening than orange-framed faces (M = 4.00), F(1,56) = 5.28, p = .025, ?p2 = .086. However, unlike for race, this difference was mitigated by counterbalancing status-color associations across participants (see below). The Race × Group interaction was non-significant in the threat ratings, F(1,56) = 0.045, p = .83. Finally, participants were significantly more confident in their racial categorizations for Black faces (M = 7.77) compared to White faces (M = 7.69), F(1,56) = 6.08, p = .017, ?p2 = .098. Confidence in racial categorization did not vary significantly as a function of group or the Race × Group interaction, both F(1,56) < 1.07, p > .30.

5. Methods: EMS and IMS Questionnaire. An example item and internal consistency estimates for each scale should be provided. This would permit, respectively, to better portray the underlying constructs, and to assess the instrument's reliability.

* We agree that this would be an important addition to the manuscript. These additional details have been added on page 10.

6. Methods: The selection of a 20-voxel cluster threshold seems arbitrary. The authors state that "This was not implemented for the purpose of statistical analysis, but rather for reporting significant clusters and their corresponding peak BSR values and coordinates within the obtained brain networks.", but this does not justify their choice. The authors should adequately control for the multiple-comparison problem, e.g., by using cluster-level correction.

* We thank the reviewer for highlighting this lack of clarity in the original submission. One of the advantages of using PLS is that it reflects the distributed co-activation of voxels throughout the brain. Because of this, PLS does not require multiple comparison correction (Krishnan et al., 2011; McIntosh & Lobaugh, 2004; Spreng et al., 2010). Unlike a traditional univariate analysis where t-tests are conducted on a voxel-by-voxel basis, behavioral PLS formally tests the degree to which a behavioral index (e.g., EMS) predicts one or more patterns (i.e., latent variables--LVs) of voxel co-activation across the brain. BSR values reflect the reliability that a given voxel contributes to the overall pattern captured by the latent variable (see McIntosh & Lobaugh, 2004), irrespective of adjacent voxels. Not surprisingly, voxels with large BSR values (positive or negative) tend to cluster with one another. This makes sense based on functional anatomy; if a brain region is co-activating with other brain regions during a task, this should presumably implicate a sizeable cluster of that region's voxels. However, this is not always the case. Indeed, there are smaller clusters of voxels that also contribute to the latent variable. Recent papers using PLS analysis tend to use tables to summarize the emerging clusters from PLS, truncating their reports at some arbitrary minimum cluster size (e.g., Cloutier et al., 2017; Lee et al., 2011; Luk et al., 2010; Martin et al., 2011; Palombo et al., 2016; Protzner et al., 2013; Spreng et al., 2010). Notably, there does not appear to be any consensus regarding minimum cluster size (e.g., Cloutier et al. use a threshold of 20 voxels; Martin et al. use a threshold of 5 voxels; Lee et al., Luk et al., Protzner et al., and Spreng et al. report effective cluster thresholds between 10-11 voxels). Based in part on this previously published work, we choose the conservative approach of focusing on relatively large clusters that contribute to the overall pattern, excluding smaller isolated clusters of voxels that would lengthen Table 1 to a considerable degree. That being said, if the editor and reviewers believe it is best to use a smaller threshold or no threshold at all (e.g., Palombo et al., 2016), we are willing to do so. Although the number of clusters is currently truncated in Table 1, we nonetheless display all voxels with BSRs greater than 2.5 in Figure 1.

7. Results: Why are the error bars in Figure 1 skewed? An explanation should be provided in the caption and/or the description of the analysis method.

* This is an important observation. Although confidence intervals are usually symmetric about their respective estimates, this is not the case for confidence intervals that are derived from bootstrap distributions (Efron & Tibshirani, 1986). We report confidence intervals derived from the standard estimation procedure built into the PLS analysis toolbox (see http://web.mit.edu/seven/src/PLS/Plscmd/pls_analysis.m). At the reviewer's suggestion, we have added further details on how confidence intervals were generated in the section on the analysis method on page 14.

8. Irrespective of how you address major concern 1, please report and briefly comment on reaction time data.

* We now briefly report on overall reaction times in the results on page 16. Details on how RTs were processed and analyzed are reported on page 11.

9. Discussion: The rostral/perigenual ACC cluster seems to be in the close vicinity or even overlapping with the vmPFC cluster originally observed in their previous mass univariate analyses. Although the authors mention this in the Introduction, they should speak to this point in Discussion. What does it mean this region emerges both here and in the original univariate analyses?

* We thank the reviewers for picking up on this point of convergence between analyses. We agree that this apparent overlap is deserving of greater discussion. We now explicitly discuss this point on page 25.

10. Discussion: the cluster in the mid-posterior cingulate, a brain region involved in introspection, deserves further comment. Although such a discussion is inevitably based on reverse inference, might this effect as well relate to self-awareness and/or reputational concerns?

* We thank the reviewers for this insightful observation and agree that this is an important point. We also agree that due caution is needed when making reverse inferences. The region in question is primarily localized to the middle cingulate, somewhat anterior to the posterior cingulate. Notably, the middle cingulate has been implicated in introspection about one's own internal states or unpleasant emotions (Doll et al., 2016; Farb et al., 2013; Herbert et al., 2011; Herwig et al., 2010). We now briefly mention this important work involving the middle cingulate in introspection, highlighting this area as a region of interest for future neuroimaging work on external motivation to respond without racial prejudice (see pages 21-22).

Minor Concerns:

1. It is not clear in the manuscript what EMS and IMS actually stand for. Externally motivated and internally motivated are easily inferred but the acronyms should be made clear.

* We agree that the meaning of the letters in these acronyms is not self-evident, particularly for researchers who are less familiar with the social psychology literature on these constructs. EMS (external motivation scale) and IMS (internal motivation scale) are derived from their respective measures, originally developed by Plant and Devine (1998). These acronyms are now clarified for the reader early in the paper on page 4. To avoid confusion, we have removed these acronyms from the abstract and significance statement.

2. On pages 5 and 6, lines 104 through 106, it is said that you anticipated any latent variables would likely implicate regions involved in person evaluation and the regulation of prejudice without providing any examples of such brain regions.

* We thank the reviewer for pointing this out. We have endeavored to add some representative brain regions for each of these processes (see page 6).

3. It would be nice to see data included regarding the explicit stimuli ratings and judgments made by participants outside the scanner.

* In addressing major concern 1e, we have now included data on relevant explicit stimuli ratings that participants made outside the scanner. We believe this was a helpful suggestion and appreciate the opportunity to revise the manuscript to report on these relevant ratings.
